# Anxiety-Related Factors Associated With Symptom Severity in Irritable Bowel Syndrome in Jazan, Saudi Arabia

**DOI:** 10.7759/cureus.53549

**Published:** 2024-02-04

**Authors:** Abdulaziz Alhazmi, Hussam Darraj, Hussain Abdali, Sultan M Hakami, Abdullah Alatiyyah, Mohammed Dalak, Khalid M Hakami, Ali Ghalibi, Hany Abdulwali, Abdulrahman M Jali, Yahya M Alawi, Shehab Hakami

**Affiliations:** 1 Pathology, College of Medicine, Faculty of Medicine, Jazan University, Jazan, SAU; 2 Surgery, College of Medicine, Faculty of Medicine, Jazan University, Jazan, SAU; 3 Medicine, College of Medicine, Faculty of Medicine, Jazan University, Jazan, SAU

**Keywords:** saudi arabia, jazan, type ibs, irritable bowel syndrome, anxiety

## Abstract

Background: This study in Jazan, Saudi Arabia aims to identify anxiety-related factors associated with symptom severity in irritable bowel syndrome (IBS). IBS is a common gastrointestinal disorder causing abdominal pain and altered bowel habits. The prevalence of IBS in Saudi Arabia is estimated to be 10%-20% among the general population. Therefore, the present study aimed to determine anxiety-related factors associated with symptom severity in irritable bowel syndrome in Jazan, Saudi Arabia. Understanding these factors will provide insights into the overall impact of IBS on patients' quality of life.

Methods: The study utilized a cross-sectional, descriptive observational design to examine the general population in Jazan, Saudi Arabia. The sample size of 385 individuals was calculated based on the population size and desired error margin. A convenience sampling technique was employed to select participants who met the inclusion criteria. A validated anonymous self-administered electronic survey was distributed through social media channels. The survey consisted of four sections gathering demographic information, personal risk factors, IBS types with anxiety and risk factors, and a questionnaire to determine the level of anxiety. A pilot study was conducted to improve the survey's clarity. Ethical considerations were followed, and data were analyzed using Statistical Package for Social Sciences (SPSS; IBM Corp., Armonk, NY, USA). Descriptive statistics and significance tests were performed.

Result: A study with 637 participants found a 31.08% prevalence of IBS, which was more common in females. Participants with IBS had a higher rate of chronic disease. There were no significant differences in demographic factors between IBS and non-IBS participants. The duration of IBS diagnosis varied, with the majority of IBS with diarrhea (IBS-D) sufferers being diagnosed five years prior. Symptom severity differed among IBS types, with those with an unspecified type reporting the highest percentage of severe symptoms. IBS types were also correlated with anxiety levels, with IBS-D sufferers reporting moderate anxiety and those with an unspecified type reporting severe anxiety.

Conclusion: A study with 637 participants found the prevalence of IBS to be 31.08%, with higher rates in females. IBS was associated with chronic diseases and higher anxiety levels. The findings emphasize the need for comprehensive management of IBS, including psychological interventions and dietary modifications, to improve patients' overall health and quality of life. Further research on genetic and modifiable risk factors is needed.

## Introduction

Irritable bowel syndrome (IBS), a functional gastrointestinal disorder, is a recognized symptom complex with abdominal pain and disturbed bowel action. It leads to a substantial reduction in the quality of life, accompanied by considerable socio-economic and psychological consequences [1]. IBS is not an uncommon chronic functional GI disorder as it affects 9%-23% of the population globally [2]. A recent systematic review and meta-analysis found that IBS prevalence was 9.2% when applying Rome III criteria and 3.8% when applying Rome IV criteria [3]. In the Kingdom of Saudi Arabia, most studies conducted on the prevalence of irritable bowel syndrome in addition to the report of the Ministry of Health estimated the prevalence at 10% to 20% among the general population of the Kingdom of Saudi Arabia, these studies used the Rome III and IV criteria [2,4-7]. The pathophysiology of IBS is not fully recognized, it is conventionally thought that IBS is a consequence of various factors comprising hypersensitivity of the bowel, change in bowel motility and inflammation and stress [8]. The absence of an accurate diagnostic tool to recognize IBS has limited its diagnosis to the use of individual medical history. Currently, the diagnosis of IBS is made by using the Rome IV criteria. The Rome IV is as follows: recurrent abdominal pain at least one day per week in the last three months, with two or more of the following factors: pain related to defecation, change in stool frequency, change in stool form or appearance. The Rome III criteria were updated to Rome IV in 2016. The Rome IV criteria have substituted "abdominal discomfort" with "abdominal pain", which is more clear and specific to patients than the vague term "discomfort" used in Rome III criteria. In addition, the abdominal pain is not necessarily relieved by defecation; it may remain the same or even increase after defecation [9]. IBS comes in multiple forms. These include IBS with constipation (IBS-C), IBS with diarrhea (IBS-D), and IBS mixed type (IBS-M)/IBS with alternating constipation and diarrhea (IBS-A). Sometimes IBS may develop as a result of an intestinal infection or diverticulitis, too. IBS-D is the most prevalent kind of irritable bowel syndrome and is known to develop when the digestive system functions more quickly than is considered normal, leading to very loose, liquid stools, also known as diarrhea. Rarely having firm stools, IBS-C is typically characterized as constipation. A 2017 study that was published in the International Journal of General Medicine found that this kind of disease affects close to 30% of persons with IBS. Both diarrhea and constipation may be present in some people with IBS symptoms. IBS is a gastrointestinal disorder characterized by recurring abdominal pain or discomfort. To better understand the different types of IBS, let's explore their definitions individually. IBS-C is characterized by symptoms such as infrequent bowel movements, difficulty passing stools, and the presence of hard or lumpy stools. Individuals with IBS-C often experience a sense of incomplete bowel movements and may have to strain during bowel movements. On the other hand, IBS-D is characterized by frequent episodes of diarrhea. Individuals with IBS-D may also experience urgency in bowel movements, where they feel the need to rush to the bathroom. Stools in IBS-D can be loose and watery, and may occur more frequently than normal, and it causes irregular bowel movements that might produce diarrhea or constipation depending on the time of day. It is very important to pay attention to the symptoms so that the doctor can accurately diagnose because knowing the type of IBS leads to better treatment measures [10,11]. There is no consensus on the etiology of IBS, but biological, psychological, and sociological factors are all believed to contribute to the onset, severity, and natural history of the disorder. Psychological distress refers to feeling anxious and depressed. These symptoms are more frequent and more intense in IBS patients, and they are associated with more gastrointestinal symptoms, disability and quality of life impairment [12]. There is a gap of knowledge considering our research topic in Jazan, Saudi Arabia. Therefore, the present study aimed to determine anxiety-related factors associated with symptom severity in irritable bowel syndrome in Jazan, Saudi Arabia. The data we will find in this regard will help to understand the impact of the disease on all domains of life on patients with IBS.

## Materials and methods

Study design, setting, and population

The research study was conducted using a cross-sectional, descriptive observational study design which was conducted among the general population in Jazan, which is located in the southwest corner of Saudi Arabia. The study targeted the general population in Jazan Province who meet our inclusion criteria; all populations living in Jazan region who agreed to participate in an online survey were included in this study. This study excluded all adolescents and children, and those who didn’t finish the survey.

Sample Size and Sampling Method

Based on the 2019 census report, the population that lives in Jazan City is estimated to be 1.673 million [13,14]. Accordingly, we used http://www.raosoft.com/ to calculate the sample size for this cross-sectional study and found it to be 385 individuals. The research study utilized a prevalence=50%, an error margin not exceeding 5%, and a confidence interval of 95% to obtain sample size. In addition, a 25% non-response rate was anticipated for this study. By using a convenience sampling technique, the sample for this study was selected.

Method and instrument of data collection

After obtaining their permission to use it, a validated anonymous self-administered electronic survey was used [12]. We distributed the electronic survey, which takes four to five minutes to complete and fill out, over social media channels, including WhatsApp, Twitter, Telegram, and Snapchat. The questionnaire is divided into four sections, each of which includes a set of questions designed to analyze different aspects that are related to a study's goals.

In the first part, age, gender, marital status, place of residence, level of education, and income were all collected. In the second section, personal risk factors including hypertension (HTN), diabetes mellitus (DM), asthma, anemia, joint pain, neurological disease, thyroid disease, psychological disease, smoking status, obesity, and physical activity compliance were recorded. The third section contains questions to assess IBS types with anxiety and risk factors. The fourth section contains 11 questions to determine level of anxiety.

Pilot Study

Before conducting the study, a pilot study including 10% of the required sample size was conducted to evaluate the participants' understanding of the survey used for data collection. Depending on the result of the pilot study, certain improvements and reorderings of some questions were made. The result of the pilot study wasn’t included in the analysis of the final data.

Ethical consideration

The study was presented primarily to Jazan University's Scientific Research Ethics Committee (REC) for review and approval. The study was approved with reference number REC-44/03/322 on 5/10/2022. This study was conducted following Saudi Arabia's ethical principles. The informed consent of each participant was acquired and obtained before starting the anonymous questionnaire. Participants were free to leave the survey at any time during the research process. No one among the participants was questioned about anything that could disclose their identity; privacy and confidentiality were maintained.

Statistical analysis

Data were verified manually by the investigators and coding was done within an Excel sheet (Microsoft, Redmond, WA, USA), then all data were entered and analyzed using the Statistical Package for Social Sciences (SPSS) version 23 (IBM Corp., Armonk, NY, USA). Descriptive statistics were calculated for study variables, e.g., frequency and percentage for qualitative variables and mean and standard deviation for quantitative variables. Tests of significance (e.g., chi-square and t-tests) were applied appropriately. A p-value <0.05 was set to indicate statistical significance.

## Results

Six hundred and thirty-seven were included in the study. The mean age was 31.16 ± 10.47 years. There were slightly more male (51.8%) than female (48.2%) participants. Nearly half were married (47.4%), while 49.3% were single. Most had a diploma or bachelor's degree (73.8%), while few had less than a high school education (2.4%). The participants' employment status varied, with 39.2% being students, 39.7% employed, 9.3% unemployed, 8% homemakers, and 3.8% retired. Regarding monthly income, 6% of participants earned less than 3000 Saudi Riyals (SAR), 15.2% made between 3001 and 5000 SAR, 26.7% earned between 5001 and 10000 SAR, 36.4% earned between 1000 and 20.000 SAR, and 15.7% earned above 20000 SAR. Based on body mass index (BMI), 39.2% had a normal weight, while 30.1% and 19.8% were overweight and obese, respectively. Forty-one percent reported no exercise per week. Nearly one-quarter (25.3%) had a chronic disease, primarily hypertension (26.1%), diabetes (23.6%), and asthma (21.1%) (Table 1).

**Table 1:** Baseline Sociodemographic Characteristics of the Participants (n = 637) SD; Standard deviation, SAR; Saudi riyal, BMI; Body mass index, Min; Minute, HTN; hypertension, DM; diabetes

Variable	Frequency	Percent
Age, years (mean:SD): 31.163±10.467		
Gender		
Male	307	51.8%
Female	330	48.2%
Marital Status		
Married	302	47.4%
Single	314	49.3%
Widowed	8	1.3%
Divorced	13	2%
Educational level		
Less than high school	15	2.4%
High school	121	19%
Diploma and Bachelor’s	470	73.8%
Master and PhD	31	4.9%
Employment status		
Student	250	39.2%
Employee	253	39.7%
Unemployed	59	9.3%
Homemaker	51	8%
Retired	24	3.8%
Monthly income		
Less than 3000 SAR	38	6%
3001-5000 SAR	97	15.2%
5001-10000	170	26.7%
10001-20000 SAR	232	36.4%
Above 20000	100	15.7%
BMI		
Underweight	69	10.8%
Normal	250	39.2%
Overweight	192	30.1%
Obese	126	19.8%
The minutes of exercise per week		
Lack exercise	261	41%
50-100 mins	197	30.9%
101-150 mins	58	9.1%
151-200 mins	52	8.2%
Above 200 mins	69	10.8%
Chronic disease		
Yes	161	25.3%
HTN	42	26.1%
DM	38	23.6%
Asthma	34	21.1%
Anemia	5	3.1%
Joint pain	27	16.8%
Neurological disease	4	2.5%

The prevalence of IBS was 31.08%. IBS was significantly more common in females than males (65.7% vs. 34.3%, P = 0.000). Participants with IBS had a significantly higher rate of chronic disease (30.3% vs. 23.2%, P = 0.037). The two groups had no significant difference in age (P = 0.245), marital status (P = 0.624), education (P = 0.585), employment status (P = 0.284), monthly income (p = 0.749), BMI (p = 0.270), or exercise (p = 0.116) (Table 2).

**Table 2:** Associations between sociodemographic characteristics and irritable bowel syndrome (IBS)

Variable	IBS					P-Value
	Participants with IBS		Participants without IBS			
	N	%	N		%	
Age, years (mean:SD)	30.44±10.056		31.487±10.64			0.245
Gender						
Male	86	34.3%	239	54.4%		0.000*
Female	130	65.7%	200	45.6%		
Marital status						
Married	92	46.5%	210	47.8%		0.624
Single	100	50.5%	214	48.7%		
Widowed	1	0.5%	7	1.6%		
Divorced	5	2.5%	8	1.8%		
Educational level						
Less than high school	4	2%	11	2.5%		0.585
High school	38	19.2%	83	18.9%		
Diploma and Bachelor’s	143	72.2%	327	74.5%		
Master and PhD	13	6.6%	18	4.1%		
Employment status						
Employee	70	35.4%	183	41.7%		0.284
Unemployed	25	12.6%	34	7.7%		
Homemaker	16	8.15	35	8%		
Retired	7	3.5%	17	3.9%		
Monthly income						
Less than 3000 SAR	14	7.1%	24	5.5%		0.749
3001-5000 SAR	29	14.6%	68	15.5%		
5001-10000 SAR	57	28.8%	113	25.7%		
10001-20000 SAR	71	35.9%	161	36.7%		
BMI						
Underweight	19	9.6%	50	11.4%		0.270
Normal	87	43.9%	163	37.1%		
Overweight	60	30.3%	132	30.1%		
Obese	32	16.2%	94	21.4%		
The minutes of exercise per week						
50-100 Mins	61	30.8%	136	31%		0.116
101-150 Mins	20	10.1%	38	8.7%		
151-200 Mins	23	11.6%	29	6.6%		
Above 200 Mins	15	7.6%	54	12.3%		
Chronic disease						
Yes	59	30.3%	102	23.2%		0.037
No	139	69.7%	337	76.8%		

The duration of the diagnosis varies across the different types of IBS. Among those with an unknown type of IBS, 15.6% had been diagnosed in less than a year, 37.5% had been diagnosed between one and five years ago, and 46.9% had been diagnosed more than five years ago. A similar distribution was observed among those with IBS-M and IBS-D. However, for those with IBS-D, the majority (62.5%) had been diagnosed five years ago. The severity of IBS symptoms also varied across types. The majority of individuals across all types reported moderate symptoms. However, among those with an unknown diagnosis type of IBS, 40.7% reported severe symptoms, which was higher than any other group. There was a noticeable correlation between the types of IBS and anxiety levels. Most individuals with all types of IBS reported moderate anxiety levels. However, those with IBS-D had the highest percentage (62.5%) of moderate anxiety, while those with an unknown diagnosed type of IBS had the highest percentage (29.6%) of severe anxiety. In terms of risk factors, stress in the last six months was the most commonly reported risk factor, particularly among those with IBS-M (71.4%) and IBS-C (56.4%). A family history of IBS was also a significant risk factor across all types. Those with IBS-D showed the highest percentage (37.5%) of smoking as a risk factor. Interestingly, those with an unknown type of IBS had the highest percentage (34.4%) of food allergies (Table 3).

**Table 3:** Comparison between irritable bowel syndrome (IBS) types with anxiety and risk factors

Variable	IBS with constipation					IBS with diarrhea				IBS with mixed type					Uknown diagnosis type				I don’t know	
	N	%				N	%			N	%				N	%			N	%
How long have you been diagnosed with IBS?																				
Less than a year	7	17.9%				2	12.5%			11	17.5%				6	22.2%			5	15.6%
5 Years	11	28.2%				10	62.5%			29	46%				13	48.1%			12	37.5%
More than 5 years	21	53.8%				4	25%			23	36.5%				8	29.6%			15	46.9%
How would you describe the severity of IBS symptoms?																				
Mild	3		7.7%			2	12.5%			4		6.3%			3	11.1%			19	10.7%
Moderate	26		66.7%			12	75%			45		71.4%			13	48.1%			20	62.5%
Severe	10		25.6%			2	12.5%			20		31.7%			8	29.6%			46	23.2%
Anxiety level																				
Normal	1			2.6%		0		0%		2			3.2%		2		7.4%		12	6.8%
Mild	1			2.6%		4		25%		7			11.1%		3		11.1%		19	10.7%
Moderate	28			70%		10		62.5%		34			54%		14		51.9%		100	56.5%
Severe	9			22.5%		2		12.5%		20			31.7%		8		29.6%		46	23.2%
Risk factor																				
Smoking	7				17.9%	6			37.5%	6				9.5%	3			11.1%	4	12.5%
Family history of IBS	15				38.5%	4			25%	21				33.3%	5			18.5%	17	53.1%
Stress in the last six months	22				56.4%	8			50%	45				71.4%	14			51.9%	12	37.5%
Allergy to some food	12				30.8%	4			25%	16				25.4%	8			29.6%	11	34.4%
Frequent use of antibiotics	4				10.3%	2			12.5%	5				7.9%	3			11.1%	1	3.1%

## Discussion

IBS, a chronic condition, is now considered a gut-brain interaction disorder [15], with many patients experiencing psychological issues [16,17]. The connection between these issues and gastrointestinal symptoms is still uncertain [18,19]. It's logical to assume that those with the most severe symptoms may also struggle with the greatest psychological challenges. However, growing evidence suggests that mood disorders can contribute to IBS symptoms [18,19]. Treatments focusing on mental well-being, such as cognitive behavioral therapy or gut-directed hypnotherapy [19,20], can help alleviate symptoms for some individuals. Therefore, it's crucial to explore the connections between general and gastrointestinal symptom-specific anxiety and the symptoms of IBS.

The prevalence of IBS in the study sample was 31.08%, slightly higher than the estimates from previous studies in Saudi Arabia that found IBS at 10-30% [4,21-25]. This may indicate that IBS is becoming more common in the Saudi population. This finding aligns with the recent study conducted in Makkah, where the prevalence was 44.9% [26]. Regarding sociodemographic characteristics, IBS was significantly more prevalent among women than men, aligning with prior research findings worldwide and in Saudi Arabia [27,28]. No significant differences were found between the IBS and non-IBS groups for other sociodemographic factors such as age, marital status, education, income, and employment. This contrasts with some studies that have reported associations between IBS and lower education or socioeconomic status [29,30]. In the present study, no significant differences in age were found between participants with and without IBS, which aligns with the existing literature [31]. The distribution of BMI among participants with and without IBS did not show significant differences. This contrasts with certain studies suggesting a relationship between obesity and IBS [32]. No significant differences were found in exercise, although most participants reported a lack of exercise. In a past study in Iran, no significant association was found between physical activity and the odds of IBS among overweight or obese individuals [33]. On the contrary, a study showed that increasing physical activity has long-lasting benefits for clinical and psychological IBS symptoms [34]. However, regular exercise is thought to improve bowel function, reduce bloating, and reduce stress, which all help to ease the symptoms of IBS [35].

Regarding health status, the IBS group had a significantly higher rate of chronic disease comorbidities than the non-IBS group. Several studies have noted high comorbidity rates between IBS, hypertension, diabetes, and asthma [36]. This is an essential consideration for the comprehensive management of IBS patients. This study found a relationship between IBS, anxiety, and related factors. A notable observation is the high prevalence of moderate to severe anxiety levels among participants diagnosed with IBS, suggesting a strong association between IBS and anxiety disorders, consistent with the existing literature [37,38]. More than half of the participants with IBS reported experiencing stress in the last six months, strengthening the known connection between psychological stressors and IBS [37]. These findings emphasize the need for a multidimensional approach to IBS management, including psychological interventions and dietary modifications. Past studies reported a prevalence of anxiety in patients with IBS ranging from 40% to 100% [39]. Studies have reported elevated levels of anxiety and depression in IBS individuals compared to healthy controls [39]. Anxiety and IBS can create a vicious cycle in which each condition worsens the other. For example, anxiety can worsen IBS symptoms and vice versa [40,41]. Anxiety is a well-known trigger for IBS, and extreme anxiety can bring on IBS symptoms [42,43]. Some evidence suggests that anxiety and IBS may share similar genetic pathways, making it more likely that a person develops both conditions [41,43]. Healthcare professionals must evaluate and treat associated psychiatric comorbidities in IBS patients to improve their overall health and quality of life. Previous studies found that about 50-90% of patients with IBS have associated psychiatric comorbidities, with anxiety disorders and depression being the most common [42,44]. A family history of IBS was also prevalent, indicating potential genetic factors that warrant further investigation [45]. Modifiable risk factors such as smoking and frequent antibiotic use were less common but still reported by a proportion of patients with IBS. The study's limitations include its reliance on self-reported data, which may lead to recall bias. The cross-sectional design also limits the ability to infer causality.

## Conclusions

The study included 637 participants, and the prevalence of IBS was found to be 31.08%. It was observed that IBS was significantly more common in females compared to males. There were no significant differences in age, marital status, education, income, employment, or BMI between the participants with IBS and those without. However, participants with IBS had a higher rate of chronic disease comorbidities compared to the non-IBS group, with hypertension, diabetes, and asthma being the most prevalent conditions. The study also examined the association between IBS and anxiety. It was found that participants with IBS had a higher prevalence of moderate to severe anxiety levels compared to those without IBS. The high prevalence of anxiety among individuals with IBS highlights the strong association between IBS and anxiety disorders.

The findings suggest that IBS is becoming more common in the Saudi population and that it disproportionately affects females. The study emphasizes the need for a multidimensional approach to managing IBS, including psychological interventions and dietary modifications. It also highlights the importance of evaluating and treating associated psychiatric comorbidities in IBS patients to improve their overall health and quality of life. The research provides valuable insights into the prevalence and characteristics of IBS in the studied population, contributing to the understanding of the gut-brain interaction in this chronic condition. Further investigations into the genetic factors and modifiable risk factors associated with IBS are warranted.
